# Statement of Retraction: Circular RNA Plasmacytoma Variant Translocation 1 (CircPVT1) knockdown ameliorates hypoxia-induced bladder fibrosis by regulating the miR-203/Suppressor of Cytokine Signaling 3 (SOCS3) signaling axis

**DOI:** 10.1080/21655979.2024.2299595

**Published:** 2024-02-20

We, the Editors and Publisher of the journal *Bioengineered*, have retracted the following article:

Teng Li, Yi Xing, Guoxian Zhang, Yan Wang, Yinsheng Wei, Lingang Cui, Shaojin Zhang & Qingwei Wang (2022) Circular RNA Plasmacytoma Variant Translocation 1 (CircPVT1) knockdown ameliorates hypoxia-induced bladder fibrosis by regulating the miR-203/Suppressor of Cytokine Signaling 3 (SOCS3) signaling axis, Bioengineered, 13:1, 1288-1303, DOI: 10.1080/21655979.2021.2001221

Since publication, significant concerns have been raised about the integrity of the data and reported results in the article. When approached for an explanation, the authors did not provide their original data or any necessary supporting information. As verifying the validity of published work is core to the integrity of the scholarly record, we are therefore retracting the article. All authors listed in this publication have been informed.

We have been informed in our decision-making by our editorial policies and the COPE guidelines.

The retracted article will remain online to maintain the scholarly record, but it will be digitally watermarked on each page as ‘Retracted’.

